# Straight from foster care to the youth detention center? The (mis)paths of child protection and juvenile justice policies in the construction of violent masculinities

**DOI:** 10.3389/fsoc.2025.1483042

**Published:** 2025-11-13

**Authors:** Marta Mascarenhas

**Affiliations:** Observatory of Masculinities, Centre for Social Studies, University of Coimbra, Coimbra, Portugal

**Keywords:** masculinities, violence, gender stereotypes, public policies, child protection, juvenile justice

## Abstract

The present article aims to understand how public policies for the protection of endangered children/youngsters and juvenile justice work together or, on the contrary, in a disjointed way, and its impacts on the reproduction of gender stereotypes, namely in the construction of rigid masculinities of youngsters in detention and the adoption of violence as an adaptive strategy. Youth Detention Centers (YDC) were chosen as a backdrop since young people at risk are a particular fringe of society who, similar to other vulnerable groups (e.g., migrants, refugees, etc.), face more significant risks of GBV, since they are displaced from their families and communities when they are admitted at Portuguese YDCs and their paths have invariably been marked by having witnessed or been exposed to violence, and often, as we shall see, to protective institutionalization at an early stage in their journey. To this end, the role of YDC's will be traced within the framework of public policies on juvenile justice in Portugal, exploring the evolution of public policies aimed at both the risk and the social danger of children and young people, namely the effects of the separation that has taken place in Portugal of these two domains in the face of the effects of the promiscuity between the domain of danger and that of social risk. Results of the X-MEN Project, namely the youngsters' narratives on their life trajectories until entering the YDC, will be used to illustrate the relationship between gender socialization (particularly of boys), the interpenetration between protective institutionalization and its predictor of a pre-delinquent path and, finally, how gender can be the basis for differentiated treatment by the systems that work with young people in danger (Child Protection System) and at risk (Juvenile Justice System).

## Introduction

The link between traditional masculinities and the intergenerational transmission of violent behavior has been empirically substantiated. Indeed, the exposure of children and young girls and boys to violence, whether in a domestic, school or community context, can lead to the normalization of violent behavior, namely gender-based violence (GBV) and a range of mental health problems, as well as influencing the use of violence as adults (Taylor et al., 2016; Till-Tentschert, 2017; Myers et al., 2018). Research has shown the number of juvenile perpetrators (aged 7–12) has grown significantly in other contexts, as well as in Portugal where, for example, there was an increase [in September 2021] of 11.11 percent, with 12 more young people in detention in comparison with the previous year. In 2022, there was the same trend, already registered in 2021, of growth in the number of young people admitted (Comissão de Acompanhamento e Fiscalização dos Centros Educativos [CAFCE], 2023, 2022).^1^ There is cause for increased concern in that these youngsters appear to be committing more serious and violent crimes (3,587 in 2022, including 4 for murder and 32 for rape), with the weight in total enquiries increasing significantly (50 percent in 2022) (Comissão de Análise Integrada da Delinquência Juvenil e da Criminalidade Violenta (CAIDJCV), 2024),^2^ and there is a tendency for them to continue on a criminal path in life (Secretaria-Geral do Sistema de Segurança Interna, 2022, 2023, 2024; Loeber et al., 2003):

Child delinquents are two to three times more likely to become serious, violent, and chronic offenders = than adolescents whose delinquent behavior begins in their teens (p. 3, 4).

Aware of this reality, this article will not only show how gender socialization, particularly of boys, contributes to their frequent adoption of aggressive and risky behaviors, leading them toward pre-delinquency, but it will also seek to explore the possible continuity of young people's trajectories from the Portuguese Child Protection System to the Juvenile Justice System, to unveil potential loopholes in these public policies that tend to be a self-fulfilling prophecy of the young person's delinquent path and future career in crime (Jouet, 2022). Furthermore, we will delve into official data from Portuguese Juvenile Justice services that sustains the possibility of a different treatment in both systems depending on the gender of the subjects.

To this end, we set out to explore the Portuguese results of Project X-MEN: Masculinities, Empathy and Non-Violence, funded by the European Union's Citizens, Equality, Rights and Values Programme, an international research-action project developed from 2022 to 2024 in Croatia, Portugal and Spain, aiming to promote non-violent masculinities and developing strategies to break cycles of violence (secondary and tertiary prevention of violence) by creating tools and interventions aimed at reducing GBV and promoting gender equality.

## Gender socialisation and violence in the construction of masculine identity

Masculinities are social constructs that define what it means to be a man. They are influenced by factors such as ethnicity, age, socioeconomic status, and cultural context (Kaufman, 1999). Rather than being innate or biologically determined, masculinities are learned through social interactions from early childhood through adolescence and into adulthood. They are also passed down from generation to generation (Schrock and Schwalbe, 2009). These constructions are embedded in social institutions, encompassing formal and informal laws, norms, and practices that define and regulate gendered behaviors. Masculinity is often associated with shared societal expectations regarding how “real” men should behave, reinforcing restrictive gender norms (OECD, 2021).

Despite multiple configurations of masculinity (Connell, 2005), dominant models continue to shape identity formation, often privileging aggression and emotional restraint. Various forms of masculinity coexist within the same institutional, cultural, and social contexts (Connell, 2005; Haywood and Mac an Ghaill, 2007), as observed in school settings where different archetypes, such as high achievers, athletes, “nerds,” and popular boys, emerge (Equimundo, 2022). Gender socialization plays a crucial role in shaping these identities, with childhood experiences influencing how individuals internalize and reproduce gendered behaviors. The normalization of violence during early socialization processes significantly predicts later aggressive behavior, reinforcing masculinity as a construct rooted in dominance and control. Young people, particularly boys in care and correctional institutions, often embody restrictive masculinities that frame their roles in opposition to femininity, perpetuating a binary gender structure and power imbalances (Connell, 2005).

Family relationships, especially parental dynamics, significantly influence children's emotional regulation and behavior patterns. Research on intergenerational violence (Ehrensaft et al., 2003) highlights that children exposed to parental conflict may learn to view violence as an acceptable way to resolve disputes (Carlson, 1990). This 20-year longitudinal study examines how childhood exposure to parental violence can affect the likelihood of individuals engaging in intimate partner violence in adulthood. The findings indicate that such exposure is linked to an increased risk of both perpetration and victimization in future relationships. This social learning process increases the likelihood of aggression in later life, including in intimate relationships (Gover et al., 2008).

Masculinism, characterized by the universalization and prioritization of harmful and restrictive masculine norms—centered on dominance, individualism, and violence—marginalizes alternative models of masculinity and social interaction (Ruxton and Burrell, 2020). Hegemonic masculinity, which fosters emotional suppression and deters vulnerability (Elliot, 2016), instills shame in men who fail to meet its rigid ideals and discourages emotional intimacy (Hanlon, 2012). Such notions of masculinity have significant repercussions for both genders, as it underpins male violence, wage disparities, and the disproportionate burden of care placed on women (United Nations Department of Economic Social Affairs, 2010).

Although dominant, hegemonic masculinity does not serve the wellbeing of men or women in contemporary societies. Studies suggest that transforming gender norms benefits men by improving physical and mental health, enhancing social relationships, and reducing violence (Connell, 2003; Hearn, 2001; Kimmel, 2010; Messner, 1997; Scambor et al., 2013). Furthermore, Messner (1997) and Kimmel (2010) argue that greater gender equality humanises men, fostering emotional wellbeing and social cohesion.

Intergenerational violence has gained increasing recognition in scientific discourse, with empirical evidence linking childhood exposure to violence with victimization and perpetration in adolescent and adult relationships (Gover et al., 2008; Chui et al., 2022). Oliveira et al. (2012) and Oliveira and Sani (2009) emphasise that family transmission of violence can emerge early in dating relationships, shaping conflict resolution strategies. Testimonies from qualitative research affirm that these patterns align with personal experiences, reinforcing the need to address intergenerational cycles of violence within the framework of gender socialization.

During adolescence and early adulthood, gendered behaviors and attitudes are often influenced and even exacerbated (especially in the case of boys) by institutional contexts, where the risk of exposure to symbolic and real violence is considerably higher, such as in youth detention centers or refugee camps. Most of these contexts are hierarchical institutions isolated from the outside world and present challenges to the promotion of empathy and equitable gender relations, since they are structured on highly stratified and vertical notions of power, which favor the existence of a dominant group and a dominated group.

In this sense, the X-MEN Project in Portugal sought to analyse how gender identities are structured in the institutional context of youth detention centers and how the affirmation of masculinity exercised by youngsters in conflict with the law explains their path until they arrive at the Youth Centers and their trajectories when they leave them. In that sense, the research was guided by the following essential question: “Why some and not others?” (Souza e Silva, 2003). In other words, the aim is to understand why these are the young people we find in the YDCs and not others, to understand what masculinities and femininities these young boys and girls are creating, how these gender identities help explain why they got there and how detention facilities soften or reinforce such social constructions.

The ultimate goals of the project were, on one hand, to understand the profile of young people currently in Youth Detention Centers (YDC) in Portugal, namely their conceptions of gender, masculinity and identity and, on the other hand, to understand how the possible absence of care in their lives cause them to adopt ways of functioning that led them to the centers.

- In the first part of this article, we will look at YDC features that can contribute to the reproduction of masculine identity models based on gender stereotypes and violence.
- In the second part, we will explore, based on the narratives of young people in detention, the correlation between a childhood marked by violence (either as a witness or as a perpetrator), the passage through the foster care system as a state child protection measure and the subsequent practice of delinquent behavior that results in sanctions from the juvenile justice system. The aim is to analyse whether the link between public policies for the protection of children and young people in danger and the juvenile justice system in Portugal contributes to the perpetuation of mechanisms for building rigid and violent masculinities, and to explore the main challenges for concerted action to break the vicious cycle of the intergenerational transmission of violence.
- Lastly, we will attempt to problematize the possibility that gender is at the root of the differentiated treatment of juvenile delinquency, resulting in a tendency for girls to be more frequently covered by the child protection system and boys to be subject to juvenile justice for similar behavior. This may indicate the existence of a more repressive view of boys and a perception of greater victimization of girls.

If such trend is to be confirmed, it affirms the need to deconstruct gender prejudices and stereotypes concerning the commission of criminal offenses, given the way they are reproduced in the juvenile justice and protection systems, to minimize the harsher and more stigmatizing nature of the State's reaction to the delinquent behavior of young men, which makes it difficult to achieve the educational and rights-promoting purposes in equitable terms that this intervention should have.

## Materials and methods

In order to gain a clearer and more comprehensive understanding of the reality of juvenile justice in Portugal, this research first sought to critically analyse recent trends and the state of implementation of public policies, as well as institutional protocols, regarding young people at risk, the services of the juvenile justice system, as well as the measures implemented during the COVID-19 lockdowns. At the same time, public policies and strategies aimed at GBV and the promotion of GE targeting young people at risk and the impact of the COVID-19 pandemic were another focus of this phase of the research.

To this end, a review of the most recent literature on the subject was carried out at national and European level, in addition to the collection and systematization of data from the annual report on internal security in Portugal (RASI), the reports of the Commission for the Monitoring and Supervision of Educational Centers, the Monthly Statistics of Educational Centers and the Annual Reports on Residential Care in Portugal and on child protection measures applied in this country.

A second moment of research took place when a needs assessment and diagnostic analysis of the young people at risk targeted was carried out in March and April 2022 in the 6 national YDCs [2 in the north—Porto and Vila do Conde; 1 in the center (Coimbra) and 3 in the center-south of Portugal—Lisbon], 4 of which were male and 2 mixed in terms of gender.

Qualitative research was needed to complement the quantitative data, to better understand the underlying social reasons and mechanisms and to uncover the specific socio-cultural issues that lead to specific masculinities and femininities and that could explain young people's journeys before delinquency. To this end, a combined methodological approach was used, which included policy mapping, quantitative and qualitative research tools. We highlight the use of three research techniques which, when combined, helped to reconstruct their trajectories from childhood to young adulthood, namely by exploring their narratives about the time before and after they entered the six Portuguese youth detention centers studied:

- Ninety-seven questionnaires, completed by youngsters (80.16 percent of the universe of young people at Portuguese YDC's at the time), made it possible to draw up a picture of the different dimensions of young people's lives in those institutions;
- Eleven individual interviews with young people (9 boys and 2 girls between the ages of 15 and 18) serving a closed detention measure (the most restrictive) which provided an insight into the trajectories, family contexts, educational paths and behaviors that led to delinquency paths, as well as 6 semi-structured interviews with YDC's Directors-−12 focus groups with young people and professionals from each of the centers. On one hand, the focus groups were spaces for young participants to share ideas and, by listening to each other's life stories, being able to reflect on their own experiences; on the other hand, the focus groups with professionals who work in the YDC's allowed us to get to know the daily reality of these professionals, their technical and psychosocial challenges, frustrations, as well as the joys they experience, despite the challenging context of dealing with young people in conflict with the law.

This combination of data collection tools made it possible to gather information on the gender-related attitudes of young adolescents at risk and their knowledge, behaviors and practices related to GBV, and how COVID-19 has impacted them. A particular focus was placed on boys and the construction of masculinities and intercultural and post-colonial approaches.

The X-MEN project was committed to upholding the ethical imperative of protecting individuals' rights and freedoms, with particular emphasis on the right to privacy, as enshrined in Directive 95/46/EC (OJEC/23.11.95). This principle is rigorously observed in all processes related to the collection, analysis, and storage of empirical data. The research team adhered strictly to national legislation and the ethical guidelines established by the Ethics Committee of CES, the host institution in Portugal. Furthermore, informed consent was duly obtained from all participants, including legal representatives in the case of minors, ensuring compliance with ethical and legal standards.

### Dilemmas caused by the structural features and institutional practices of youth detention centers in perpetuating violent models of masculinity

Youth Detention Centers in Portugal foster a context that, despite advocating in its legal framework aims of re-socialization and education to become law-abiding citizens as its primary goals, consists of a highly structured and mostly male environment (in a ratio of 9 to 1, in Portugal—Comissão de Acompanhamento e Fiscalização dos Centros Educativos [CAFCE], 2023). In April 2024, out of a total of 141 young people admitted, 118 (83.69%) were male and 96 young people were aged between 16 and 20 years old (Comissão de Acompanhamento e Fiscalização dos Centros Educativos [CAFCE], 2024), and, as such, the models and practices of the young detainees often reflect rigid conceptions of masculinity and, more specifically, aggressive masculinity.

One of the first characteristics we can identify in these contexts is the gap between their legal purpose, which is pedagogical, and their institutional practices, which are based on a disciplinary and often punitive logic.

Similarly to other European legal frameworks, the Tutelary Educational Act (TEA), (Law No. 166/1999 of 14 September),^3^ which regulates the juvenile justice system applied to young people between the ages of 12 and 16 who commit acts classified by Portuguese criminal law as criminal offenses, determines the purpose of its intervention as being to rehabilitate young people with a view to educating them for a life in society that complies with the law (Santos et al., 2010). This need for legal re-education is one of the mandatory requirements that must be met for the application of an internment measure to be legitimate, however this pedagogical nature seems to be ignored even within the judicial system:

The Youth Detention Center can no longer no longer be considered a prison for the little ones, but rather the last stronghold of the young person's right and the possibility to change their life, with the necessary support to change their lives (Comissão de Acompanhamento e Fiscalização dos Centros Educativos [CAFCE], 2023).

It is based on a pedagogy of responsibility, however, when you compare this legal purpose with institutional practices in YDC's, there is a mismatch between these two dimensions, since YDCs adopt very rigid quasi-prison characteristics, creating an artificial environment in which children and young people acquire routines and norms, but often do not willingly comply with them, but merely follow them as a survival strategy. As Zoettl (2017) emphasizes,

While the law gives priority to young offenders' “education,” both the sentencing practices of the judiciary and the institutional practices of YDCs adopt and implement primarily a disciplinary stance on youth justice (p. 490).

In the conclusions of its 2024 report, the Commission for the Integrated Analysis of Juvenile Delinquency and Violent Crime (CIAJDVC) stresses that the misinterpretation of the educational vs. punitive nature of juvenile justice has an effect on the actions of the authorities themselves, since “frontline organizations are reluctant to report offenses committed by young people… they see the Juvenile Justice Process as a “punishment” and not as an opportunity that can be given to young people” (2024, p. 12).

## Daily routine and permanent monitoring as tools for socio-educational intervention

Although their previous lives were, in most cases, marked by violence, fear, and suffering, according to data from the Directorate-General for Reintegration and Prison Services (Directorate-General for Reintegration Prison Services (DGRSP), 2024, 2023, 2022, 2021), they were also characterised by immense freedom and a lack of rules and supervision. This resulted in a stark contrast with the institutional reality they encountered upon entering the YDC.

A number of the youths had dropped out of school (which was described as “a big bore”), and parental and institutional authority was often minimal or completely absent. Finding themselves suddenly within the confines of a YDC, and being obliged, under threat of punishment, to obey an extensive catalog of minute rules and regulations, at first constituted a traumatic experience for the majority (Zoettl, 2017, p. 499).

In such a strongly hierarchical system, based on constant surveillance and monitoring, resistances are created, leading youngsters to adopt behaviors that may allow them to rise in an informal hierarchy among their peers. In such background, young boys (and fewer girls) often resort to violence as a strategy of affirmation within the YDC's walls, mimicking behaviors learned not only in their socialization process, but also, and often, in the institutional contexts they have experienced throughout in their short lives, namely in foster care, in which they lived prior to being detained, where they should have been specially protected by the State.

As confirmed in the 6 focus groups held with professionals from the Portuguese YDC's, all of them, without exception, identify and use the programming of daily routines as a working tool, with the aim of contributing to the mental structuring of young people (Marteleira, 2011). Thus, they recognize that the most effective way to get these youngsters, most of whom come from very unstructured backgrounds, to acquire habits and routines is to make them compulsory in the YDC:

Undoubtedly, a structuring factor for these young people is changing their daily lives so that they integrate values into their routines (Professional A, YDC1).

- Such model of intervention is reinforced by the establishment of an Incentives and Earned Privileges System, that determines different stages of access to certain privileges or, on the contrary, the application of sanctions or, in the most serious cases, referral to the courts based on their compliance with the rules and their demonstration of institutionally acceptable behavior (Cox, 2011).
- This is what (Zoettl 2017) identifies as the “carrot and stick” approach in describing the center's everyday routine. This is a common practice in other juvenile justice systems, and aims to promote self-accountability in young people, making them understand that any attempt to escape responsibility for their actions by externalizing guilt will result in a setback in their rehabilitation process, by shifting their process of change to an external locus of control (Cox, 2011):

All the facilities operated an Incentives and Earned Privileges Scheme, in which young people ascended “stages” based on their compliance with the rules and their demonstration of institutionally acceptable behavior (…). A resident successfully ascended a stage by demonstrating that they could take responsibility for their actions and by showing that their goals and behaviors were self-motivated (p. 596).

In each Portuguese YDC, the execution of internment measures is governed by a system of four progressive stages that all young people are familiar with and which structure the set of benefits and penalties they suffer during their stay in the institution:—the Integration stage (stage 1), the Acquisition stage (stage 2), the Consolidation stage (stage 3) and the Autonomy phase (stage 4):

No, because here it works in phases, in phase 1 it's integration and you can only call your direct relatives, father, mother, siblings and you can't visit boyfriends, you can't visit friends or anything, but then you go to phase 2 and in phase 2 it's already acquisition when you start to get more things, I've only been here five months, I've only been here a short time, so I can call her, but next month I'll be able to visit her, but for now I can't visit her (Young boy 2, YDC1).

To track the progress of young individuals through different phases, they are evaluated on a scale from 1 to 5. This evaluation can lead to various consequences, such as changes in curfew or the opportunity to go home for the weekend if they are serving an open or semi-open sentence. The assessment takes into account the length of their stay and their demonstration of pro-social skills, which enables them to gain greater freedom and autonomy based on how well they have met their personal educational goals (in accordance with Article 12 of Decree-Law no. 323-D/2000 of 20 December, General and Disciplinary Regulations for Youth Detention Centres [GDRYDC], 2000). To implement this system, the behavior of the young individuals is monitored on a daily and weekly basis by technical tutors. Youngsters are encouraged to reflect on their experiences and collaboratively seek solutions to daily challenges. This approach serves as an effective strategy to help young detainees understand the importance of following rules and practices, which they are hoped to take into their future lives beyond the walls of the YDC (reward and punishment philosophy). Scores of one or zero, in addition to determining the non-progression or regression of the stage, will imply disciplinary consequences, either by aggravating the legal measure applied, in accordance to TEA, or by isolation in the YDC, entailing loss of privileges:

To react? There are punishments here. If I'm at an advanced stage, I regress and I'm also locked in my room to reflect. (Young boy, YDC2)

Although there are many studies on the characteristics and impact of different models of juvenile justice (Carvalho, 2017; Santos et al., 2010; Pedroso et al., 2010), it is important to understand how the strategies and practices used by YDCs can hinder the law-abiding education purpose of the system as a whole.

It is suggested that the regime of confinement and strategies of reward and punishment of the YDCs induce juveniles to adopt only short-lived changes of conduct, ultimately confining the law's precept of education to a praxis of behavioral conditioning (Zoettl, 2017, p. 490).

Like other seemingly innocuous rules that govern daily life in the centers, this system of stages, despite its character of self-responsibility, has the perverse effect of creating a system based on the fear of permanent punishment and competition. The adoption of behaviors that are considered socially desirable occurs not so much because of an actual adherence to the pro-social values that are being promoted, but rather because of the fear that if they don't adopt them, even if only in that time and space, they may be subjected to additional restrictions in a context that is already highly restrictive of their freedom and autonomy.

This compliance with the rules due to fear of reprisals is particularly pronounced in the post-pandemic phase, when the X-MEN investigation took place and all the young people, due to the lockdown, served their internment measures under closed conditions, completely deprived of contact with the outside world. This is the basis of youngsters' behavioral change and, as such, its sustainability when the young person goes outside becomes exponentially less (Zoettl, 2017).

## Contradictions of the system: the challenges of promoting personal autonomy in contexts of restricted freedom

Most of the participants in the X-MEN research who answered the questionnaire were between 16 and 17 years old; 90% said they were male, 9% female, and 1% other. They are mostly young people born in Portugal, but there are also those born in Brazil, Cape Verde, Mozambique and Angola (as well as other nationalities, in smaller numbers) and they are resuming their studies at the YDCs, generally attending the 7th, 8^th^, and 9th grades, which reveals a clear gap between their age and the level of schooling they are attending [these findings are based on data collected through the X-MEN questionnaire (personal communication, July 2022)].

According to the results of the study, Portuguese young people believe they have good mental health (53 percent described it as very good and 29 percent as good). However, when this issue is explored in depth in individual interviews with both young people and professionals, there is a clear tendency toward medicalization as a strategy to control young people described as suffering from multiple emotional and affective needs and consequent mental health challenges. More than 60 percent take medication on a regular basis (Caruso et al., 2023) and issues such as anguish, depression, loneliness and the continuous use of calming and anxiety-controlling medication emerged as challenges faced before and during their stay in the YDC.

## Another challenge to promoting personal autonomy and agency in contexts where freedom is restricted concerns issues relating to young people's clothing and image

The General and Disciplinary Regulations for Youth Detention Centers, published in 2000 and never amended since then, contain specific provisions regarding the clothing and footwear that young people can wear in these establishments. Article 61 establishes the general principle that young people should wear their own clothing and footwear, ensuring that their use does not conflict with the execution of training activities and with current social norms, and provided that this is in accordance with the internal regulations of each YDC. This margin of discretion granted to each center through their internal regulations has made it impossible to apply this measure in practice for a long time.

Taking the case of YDC5 as a practical example, before 2019, all young people who were admitted to the YDC had to have a short haircut (comb 4) and were obligated to wear only clothes provided by the center. In 2019, less proximity to the logic of depersonalization typical of the prison regime, the YDC changed its internal regulations, revising these provisions: it is now accepted that young people can wear personal clothing at the center, can wear their hair as they wish, as long as it is kept in hygienic conditions, and can have their own personal hygiene products.

This change, apparently totally positive, is an example of how seemingly innocuous rules can have contradictory effects: if, on the one hand, the freedom to wear their own clothes allows young people to maintain an increased level of comfort and their uniqueness, what we saw in practice was that this was used to establish mechanisms of differentiation and a form of affirmation of power relations, particularly among young men. This does not seem particularly surprising when we consider the sociability of teenagers and young people who assert themselves, in terms of identity, by sharing a common aesthetic, and Portugal's Educational Centers are no exception, so we came across young people wearing jeans, T-shirts and jackets from famous “brands”.

In this way, the reason for the delinquent activity of some of these young people is imported into the walls of the YDC—this is the case of Mário (fictitious name), a young man serving time at YDC6, who says that he did not lack the basics, which were always provided for him by his grandmother. However, clothes, mobile phones and money were part of his desires… “that illusion,” in his words (Mário, X-MEN Participant, personal interview transcript, 2022). This can be particularly problematic when young people from different gangs meet in the same center, increasing the risk of violence and externalizing manifestations of power with displays of wealth (e.g., money, expensive clothes).

## Life trajectories marked by adversity and violence

Another area where the immanent contradiction of the system can be seen is in cases where the refractions of violence experienced by young people are not taken into account in the YDC or when violence is used as a strategy to control young detainees. Drawing on the notion of (Bartlett and Sacks 2019, n.p.), we understand childhood adversity as “a wide range of circumstances or events that pose a serious threat to a child's physical or psychological wellbeing,” including “child abuse or neglect, domestic violence, bullying, serious accidents or injuries, discrimination, extreme poverty, and community violence.” Given the profile of the young people in Portuguese Youth Detention Centers, responses to the X-MEN questionnaire highlight common patterns in their life stories. These include exposure to domestic violence, the absence of a father figure, and upbringing in socioeconomically disadvantaged areas marked by poverty and social exclusion. Forty-nine percent of the youngsters reported daily drug use (X-MEN questionnaire data, personal communication, April 2022).

A significant proportion of respondents (73%) spent part of their childhood and adolescence in foster care, a circumstance that often exacerbates emotional ruptures, relational distortions and a feeling of abandonment.

This is true for Maria (a fictitious name), a seventeen-year-old girl residing at YDC5 for the past 8 months, located 278 km away from the city where she used to live with her family. Her parents are Portuguese, and they have been divorced since she was 4 years old. Maria has an older brother who lives in the Netherlands, and her relationship with her mother has always been highly strained, characterized by frequent physical aggression since her childhood. As a teenager, Maria discovered that the man she believed to be her father was not her biological parent. This revelation had devastating consequences: it intensified conflicts with her mother, led to regular physical abuse, decreased her interest in school, resulted in academic failures, and marked the beginning of her drug use and delinquent behavior. This ongoing cycle of conflict ultimately prompted her mother to decide to send Maria to a foster home, starting her journey within the System (Maria, X-MEN Participant, personal interview transcript, 2022).

This sense of abandonment and invisibility extends beyond the family to society as a whole and persists even after young people enter the YDC. As one professional from YDC3 stated during a focus group discussion, “*No one wants these kids back!”* (Focus Group of YDC3 Professionals, 2022). This striking remark underscores a harsh reality: even when these individuals express a willingness to continue their education after leaving the YDC, public schools often refuse to enroll them. Moreover, they encounter significant barriers to employment, as institutions frequently view those who have undergone tutelary educational measures with suspicion, failing to recognize their potential.

The professionals have dedicated considerable effort to support the youth during their time at the YDC. A pattern of delinquency is evident in the life trajectories of the family members and friends of these young people. For example, Mário shares the story of his cousins, highlighting the similarities in their experiences: involvement in theft and robbery, a shared passion for football, time spent in youth detention centers, and the fact that they also became teenage parents while serving their sentences (Mário, X-MEN Participant, personal interview transcript, 2022).

According to the preamble of the Portugal (2000), the educational purpose of the intervention should be emphasized, characterised as “a phased and progressive process in which the disciplinary regime should function as an instrument of last resort to correct behavior for which pedagogical actions do not prove sufficient” (Portugal, 2000, n.p.). However, this subsidiary nature of disciplinary action, as stated in the legal text, comes up against institutional practices, particularly in the absence of specialized resources and training for professionals, which means that manifestations of trauma and other mental health problems resulting from the frequent adverse childhood experiences that these young people have are perceived as disciplinary issues or deviant behavior. Of the entire universe interviewed, 82% of youngsters at Portuguese YDCs are currently serving time in a closed regime, the most restrictive of all, which reflects the practice of serious offenses.

The connection between young men and women's exposure to various forms of violence and its intergenerational transmission is now well established. This exposure leads to a higher likelihood of aggression and the perpetuation of violence (Moura et al., 2024d; Belghiti-Mahut et al., 2012). According to Miller (2014), young individuals with a history of trauma who follow a criminal trajectory in adulthood often share a range of environmental and neuropsychological factors, including low verbal IQ, impulsivity, and substance abuse, among others. Although not all children with a history of adversity experience trauma or symptoms related to post-traumatic stress (Michiels, 2024; Bartlett, 2020; Copeland et al., 2007), studies show the potential that detention institutions, such as youth detention centers, have for reinforcing trauma, which leads them to experience adversity as psychologically or physically threatening or harmful (Jamieson, 2020; Bartlett and Sacks, 2019):

Pickens (2016) says that when a traumatized young person enters a youth detention center for the first time s/he often experiences a strong sense of hyper-vigilance. This is due to their usual sense of danger being further heightened by finding him/herself in an unfamiliar environment, coupled with their ongoing struggles to minimize any trauma reminders. (Jamieson, 2020, n.p.)

The past of frequent exposure to violence in early stages of life tends to translate into two risks for the youngsters' rehabilitation: the normalisation of violent behavior, which leads them to incorporate violence as an appropriate response to any feeling of threat they experience (real or imagined) and, in this sense, some emotional apathy and affective blunting in relation to the consequences of their own violent behavior, on the one hand; and, on the other hand, and in direct correlation with the previous dimension, the worsening of the likelihood that they will adopt violent behavior (Slegh et al., 2021; Jamieson, 2020), reinforcing the intergenerational transmission of violent behavior through its normalization by reference figures in the course of their childhood socialization process. The context of youth detention centers, naturally perceived as threatening, especially in the initial phase of confinement, can lead young people to respond with physical aggression or, conversely, physical and emotional distancing from others in an attempt to keep themselves safe (Jamieson, 2020).

Advocates of trauma-based approaches emphasize that young people who have experienced trauma should not be blamed for their victimization but instead bear the burden of its psychological consequences (Joiner and Buttell, 2018; Pickens, 2016). To cope with this distress, they often develop survival strategies to suppress traumatic memories and the associated pain (Shapiro, 2018).

Trauma impacts individuals across three key domains: safety, which refers to the need for protection both during and after traumatic events; control, as trauma often involves a profound loss of agency; and responsibility, where individuals may internalize blame, negatively affecting their self-esteem. When these domains are triggered in young people, they exhibit heightened needs for predictability, security, control, and non-judgment.

Each domain elicits specific emotional responses, underscoring the crucial role of professionals in intervening accordingly:

- When fear (linked to safety) is triggered, it is crucial to provide immediate reassurance.
- Sadness, guilt, and shame (linked to responsibility and self-esteem) require messages of self-compassion and externalizing blame for the traumatic event.
- Addressing anger (linked to control) by fostering a sense of agency and empowerment is a key responsibility of trauma-informed professionals.

Since boys are socialized to suppress vulnerability associated with fear and sadness, anger often becomes their dominant emotional response (Pollack, 1998). Trauma-informed professionals recognize how institutional procedures may act as triggers and, therefore, adopt strength-based approaches that enhance resilience and mental wellbeing (Malvaso et al., 2024; Flocks et al., 2017) which has a significant impact on the emotional responses of young people.

In light of the above, and considering the lack of specialized resources in YDCs (Comissão de Análise Integrada da Delinquência Juvenil e da Criminalidade Violenta (CAIDJCV), 2024; Comissão de Acompanhamento e Fiscalização dos Centros Educativos [CAFCE], 2023), it is clear that their professionals need to receive ongoing training in order to adopt a masculinity-informed trauma lens when working with at-risk youth (Michiels, 2024; Miller, 2014). This will allow them to better understand the gender-specific aspects of trauma, namely the coping responses of men and boys, as well as the links to salient masculine norms, considering the underestimation of male trauma (Slegh et al., 2021, p. 10). In this way, it can be avoided that such behaviors are seen and repressed by these professionals as disturbances or intentional displays of disobedience, helping these professionals to address them appropriately (Michiels, 2024).

Trauma-informed professionals at YDCs understand the potential and implications of some of the centre's procedures to trigger these youngsters and can adopt a strengths approach that promotes their resilience and mental health (Flocks et al., 2017), namely:

For instance, physical or mechanical restraints used in youth detention centers can be trigger a reaction of panic and flashbacks (Jamieson, 2020, n.p).

Even more serious contradictions in this area are the episodes of occult violence against young people, who receive a formal message condemning violence and are surreptitiously targeted, which acts as a message reinforcing the exercise of violence as legitimate.

Studies such as that by Belghiti-Mahut et al. (2012) have shown that violence is frequent in adolescence, and that the risk of its occurrence increases exponentially in institutional contexts where boys and young men are found, such as YDCs or prisons, due to their remoteness and society's usual disregard for what goes on in these contexts. These are also contexts where young people come together with paths marked by adversity, trauma and vulnerability and, as we saw earlier, this condition, combined with the fear caused by institutionalisation, results in the frequent use of aggressive and violent behavior, especially by boys, as an adaptation strategy and an attempt to regain some control over their new reality. This is the case for most of the individuals in Portuguese YDC's. As the Director of YDC1 points out:

Society is increasingly confronted with new forms of violence and an expanding range of channels for its perpetuation. Beyond domestic violence, corporal punishment, and violence within communities and schools, there is a growing concern over the early exposure of young people to digital violence—an omnipresent, unregulated space that can amplify both individual and collective grievances. While digital platforms offer unprecedented opportunities for learning and knowledge acquisition, they also facilitate new forms of exposure to violence, including the dissemination of radical ideologies and the potential affiliation with pro-violence groups and most of our boys are keen to use them widely. (Director of YDC1, X-MEN Participant, personal interview transcript, 2022).

The systemic contradictions emerge when institutional practices within the YDC legitimize this recourse to violence, particularly when we refer to a phenomenon of hidden violence that results from constant micro-punishments by its professionals on young people.

The offenses and disciplinary measures are exhaustively listed in the TEA and the GDRYDC, which speaks, contrary to what should be the exclusively pedagogical emphasis of these measures, to the idea of punishment (under Article 189(3) of the TEA). However, as Zoettl (2017) points out, the state system is based on the idea of incentives and privileges and the daily life of the center is marked by constant micro-punishments:

The YDCs' institutional routine, however, is imbued with the “microphysics” of disciplinary power and is clasped by a web of “infra” or “micro-penalities” (Foucault, 1979, p. 178). These penalties constitute their chief modus operandi, which is held together by the centers elaborate stage and token system (Zoettl, 2017, p. 504).

Given the normalization of violence to which most of these young people have been exposed since childhood, this logic of permanent punishment, combined with the possibility of physical restraint as “the use of physical force to immobilize and eventually remove the student” (Article 90, TEA) and precautionary isolation, reinforces their vision of a world in which the use of violence is not only a legitimate strategy, but also an efficient one for resolving conflicts and asserting oneself.

The COVID-19 pandemic has exacerbated some of these practices, particularly with regard to the isolation of young people in YDCs, since, from 2020 to 2023, every youngster admitted to any YDC in the country had to spend an initial 14 days in a Prophylactic Isolation Unit (one center for girls and one center for boys), which, according to the professionals interviewed at X_MEN, exacerbated fear, isolation and other symptoms such as anxiety, sleep disorders, depression, etc. (Focus Group of X-MEN Professionals—YDCs 1, 2 and 4). Here we have two intertwined issues that transform the experience of these young people at the specific moment of the pandemic: the ritual of moving from freedom to internment, aggravated by the fact that they were totally isolated before they knew which center they were going to, whether near their family, near or far from their place of origin.

Although containment measures can only be used in legally specified and exhaustively numbered situations, and, in the case of precautionary isolation, they must not last longer than 24 h [Article 183(2), TEA], this doesn't always seem to be the case, particularly when young people display unruly behavior or manifestations of mental suffering, in a discretionary interpretation of the legal justification for the use of physical containment measures, namely “To overcome the violent resistance of minors to the orders and instructions of the center's staff in the legitimate exercise of their duties” [Article 179, n°.1(d), TEA].

This set of contradictions to which young people are subjected when they enter YDCs reflects the challenges to their personal agency and the philosophy of normalization that seems to preside over educational tutelage intervention, reinforced above all in its institutional practices, namely the use of medicalisation and prerogatives of containment as strategies to maintain institutional functioning to the detriment of young people's autonomy and identity, which seems to be secondary to their condition as inmates (Caruso et al., 2023; Zoettl, 2017; Cox, 2011).

These are the challenges that young people face to adapt in “total institutions” (Goffman, 1961) such as YDCs, where a specific set of people, in this case, youngsters who have committed criminal offenses, are removed from society for a certain period of time, sharing the same space and having their daily activities and routines administered by a force of authority, as we have seen when talking about the use of daily routines as a social intervention strategy:

(…) all phases of daily activities are carefully planned, with one activity leading into the next at a prearranged time and often to meet organizational rather than individual needs (Goodman, 2013, p. 82).

In these contexts, young people detained in YDCs are somehow “forced” to comply with institutional regulations and orders by the permanent fear of punishment and loss of privileges, placing them in a limbo between the imposition of a highly structured and controlled routine and the need to demonstrate adequate personal autonomy (Gonçalves, 2008). It is therefore important to reflect on how the bureaucratic control character of the centers clashes with the need to promote positive learning for young people in terms of their autonomy and respect for their personal dignity (Mihaila et al., 2018), instead of favoring a paternalistic and condescending emphasis on institutional practices to standardize behavior—the idea of *mortification of the self* (Goffman, 1961), generating disparate reactions that alternate intermittently between the creation of an artificially compliant or defiant persona (Goodman, 2013; Cox, 2011).

In conclusion, it is essential to promote not only adaptation, but also young people's capacity for effective action, even in total institutions, and for this to happen, juvenile detention centers must find a better balance between the need for control and respect for young people's free will, instead of comforting young people with “what Halsey called ‘utterly incongruous demands' on young offenders in custody, such as developing ‘self-responsibility for their future despite not being allowed to decide when they go to the toilet, when they eat or when they sleep”' (2007, *cit. in* Zoettl, 2017, p. 505).

### The journey from child protection to juvenile justice—The contradictions of public policies for the protection and rehabilitation of youth at risk

Systemic changes that have taken place within the framework of public child protection and juvenile justice policies in Portugal must be analyzed, to better understand why children who are removed from their families to be protected by the State are more likely to adopt delinquent behaviors. Until the legislative reform of 1999, the Portuguese State treated both endangered and children and young people at risk more or less indiscriminately, with the understanding that youngsters who adopted deviant behavior should be considered as needing protection. On the other hand, the current legal framework is based on separating the systems and spaces dedicated to children/young people in danger (Child Protection System) from situations of risk (Juvenile Justice System), as a strategy for guaranteeing their fundamental human rights. More than 20 years on from this reform, in which the promotion of the rights and protection of endangered children and young people and intervention with children at risk in Portugal involves, depending on the nature of the situations (danger or risk) and the age group concerned, local or community intervention at first level, subsidiary intervention by the Child Protection System and, in the case of risk, parallel or exclusive intervention by the Juvenile Justice System, we now seek to understand how the transition from a single, undifferentiated system for dealing with children in danger and at risk, to the current configuration of two autonomous systems, can be understood as a factor in rigidifying the construction of masculinities and the dissemination and perpetuation of phenomena of youth violence.

The Portuguese juvenile justice system differs from most other EU countries, giving less importance to the offense than to the need for the offender to be educated on the fundamental community values that have been violated by the illicit act. It can be regarded as a third perspective falling in between a welfare model and a punitive or penal one (Carvalho, 2014, p. 3).

The collected data appears to support the previously identified trend (Santos and Rolino, 2020) regarding the differing pathways based on gender when young individuals engage in similar illegal behaviors. Specifically, the findings indicate that young females typically make their first contact with the system through a Child Protection Order; In contrast, young males usually encounter the system through an Educational Guardianship Measure. On April 1, 2021, for example, there were 99 young people in foster care in Portugal. Among these individuals, 80.8% were involved in a child protection case. Only 43 of these young people were living with their family of origin, while the remaining 56 were placed in foster care under a child protection order [Youth Detention Centres Monitoring and Inspection Commission (CAFCE), 2021].

From analysing X-MEN's quantitative and qualitative data, it seems possible to identify a pre-delinquency pathway that includes a previous entry into the child protection system. At the time of the study, more than 90% of the young people interviewed was involved a previous child protection case and had predominantly been in foster care, with many of them starting to commit offenses precisely when they entered foster care, where they learnt these delinquent behaviors.

This is the case of Maria. Entering foster care, and the sense of “freedom” she described, enabled her to associate with older individuals, experiment with several substances, and become involved in criminal behaviour. In her own words:

*I only smoked hashish, cannabis… Then, when I went to the institution, I began to discover other drugs. That's when I started hanging out with other people, people older than me, and when I came to Covilhã and as they were friends and a town close to each other and already knew each other, I started hanging out with older people from Covilhã and Castelo Branco and that's when I discovered other drugs and that's when I committed offences. I stole my mum's credit card to run away to Porto with my ex-boyfriend and a friend of mine from the institution and we went to Porto and then, when I came back, I broke some things in the institution and that was another process and then I went to a therapeutic community, to see if I could cure myself, and I went to the Alentejo, very far away, it was closed, I couldn't go out for anything* (Maria, X-MEN Participant, personal interview transcript, 2022, YDC5).

The trajectories of these young people are clearly shaped by the implementation of Child Protection and Welfare Measures before they enter into youth detention centers. This indicates a prior—albeit unsuccessful—interaction with the Portuguese National Child Protection System. Among the respondents, 64% reported having been subject to more than one child protection measure throughout their lives. In other words, nearly two-thirds of the surveyed population have experienced institutionalization, meaning they have been under state care from an early age due to their condition of vulnerability, as stipulated by Portuguese law.

This data allows us to make a first attempt at tracing what appears to be the systemic journey of these young people to the youth detention center, taking this opportunity to present the essential dimensions or levels of their profile:

- Starting from a childhood marked by family dysfunction and exposure to violence and, in the case of boys, aggravated by a gender socialization that “pushes” them toward an ideal of stoic, brave and aggressive masculinity, which reinforces the normalization of violence (individual and family levels); in a second phase, these children and families are monitored by the Child Protection System and, in the most serious cases, children are removed from their original family environment and institutionalized. According to the youngsters' accounts, it is often in these institutions that they come into contact with delinquent practices and, at the same time, are even more exposed to peer group pressure (Haynie, 2002), trying to display the traits of their dominant masculinity (interpersonal/relational and institutional Level); in the face of this trajectory, punctuated by school failure and deviant practices, young people, usually from more impoverished territories (and, consequently, with excessive policing), commit criminal offenses and end up at YDCs, where, despite their basic pedagogical philosophy, they are exposed to punitive intervention models in a context that often enhances the trauma resulting from the adversities that marked their adverse childhoods (Institutional Violence). Their passage through the juvenile justice system leaves them “scarred” and hinders their effective social reintegration and an adaptive educational and professional path after leaving the center, making recidivism more likely.

In order to guarantee the safety and wellbeing of endangered children and youngsters, namely due to exposure to domestic violence, the Portuguese State ensures that protection measures are applied and can even remove a child/youngster from their family environment and arrange for them to be placed in foster homes within the scope of the Child Protection System (Child Protection Law no. 147/99, of 1 September).^4^ According to CAFCE data, of the 99 young people in residential care in Portugal on 1 April 2021, 80.8% had a child protection process underway, with only 43 young people living with their family of origin, while the remaining 56 had a promotion and protection measure in foster care.

X-MEN research revealed that young people's journeys in the YDCs are marked by the application of these protection measures before (or at the same time as) being referred to the Juvenile Justice System. In other words, most of the youngsters surveyed had previously (and unsuccessfully) been through the National Child Protection System: around 2/3 of the youngsters at Portuguese YDCs in 2022 had been in foster families (in the care of the state from an early age) before being detained. This previous family instability characterizes, for example, the life of Luís (fictitious name), a sixteen-year-old boy being held at YDC1, for example. The son of a Portuguese mother and a Cape Verdean father, he says he knew nothing about his father:

(…) since I was little, since I was two years old, I lived with my grandmother, my aunt, my cousin, because when my mother got pregnant, she was 14 and she went to those Shelters and then she stayed there for a while, until I was born and then she left and gave me to my grandmother. (Luís, X-MEN Participant, personal interview transcript, 2022).

Of all the young people surveyed, 64 percent said they had been subject to one or more child protection measures throughout their lives. In other words, almost two thirds of the youngsters had been institutionalized, i.e., we are dealing with children and young people whose childhood was affected by extreme adverse experiences and who, from a very early age, have been under the authority and (alleged) protection of the state (Mascarenhas, 2024). Due to this pattern of adversity, at an early stage of development, the risk of more serious sequelae arising from these experiences is significantly exacerbated (Bartlett and Sacks, 2019; Bartlett, 2020; Felitti et al., 1998).

This is the case of Bruno (fictitious name), a 17-year-old boy born with withdrawal syndrome because of his mother's drug use during pregnancy. He has seven siblings, but only knows two of them. Raised by his Cape Verdean grandmother and Portuguese grandfather, Bruno first met his mother when he was 6 years old. It wasn't until he was 6 that he had his first contact with his mother. He recounts that he began dealing drugs at the age of seven in order to support his family.

When I was seven, I started noticing my grandparents arguing over money. There just wasn't any. So, I went for the easiest way out—the one where people actually accepted me—drug dealing. And that's how I got into it (Bruno, X-MEN Participant, personal interview transcript, 2022).

In Bruno's case, his itinerancy in foster homes began at the age of 9, after his grandmother reported him to the public authorities to protect him when she realized he was dealing drugs. Between the ages of 9 and 11 he had already been in at least two foster homes. And, like the other 10 young people interviewed, he reported that it was in the foster care system that he learnt to use violence, theft, among other deviant practices (X-MEN Interviews, 2022). Although adverse childhood experiences do not necessarily equate to a bad outcome and most children, properly supported, can overcome them, nevertheless, because they occur at structural and sensitive stages of human development, they can lead to physical and mental health problems in adulthood, especially in children removed from their families early in life (Bartlett and Sacks, 2019; Felitti et al., 1998).

It is therefore this bumpy road that turns the vulnerable and endangered children into young delinquents who are more likely to end up in prison. It is, therefore, relevant to consider how public policies, especially Child Protection and Juvenile Justice, and their (lack of) articulation, can perpetuate harmful models of masculinity, reinforcing the determinism of the cycle of intergenerational transmission of violence and sending young people on a fast track from home to prison. North American literature, for example, highlights the existence of a “*school to prison pipeline*,” analyzed by various authors (Marchbanks et al., 2018; Na and Gottfredson, 2013; Theriot, 2009; Kim et al., 2010; Kupchik, 2010) to designate the way in which the combination of factors such as precarious schooling, associated with exclusionary disciplinary practices; the constant presence of the police at school, and changes in “juvenile delinquency” laws as determining factors for the constitution of a *path* that leads them directly from school to prison (Rossato, 2002).

It should be noted, however, that the data collected shows that both the conditioning toward a deviant path and the specific way in which the public authorities respond to it refer to a specific profile of young people determined by the intersectionality between gender, race and socio-economic status: in the Portuguese context, it materializes in poor young people on the outskirts of cities, with young people of African descent and Roma being the most common ethnic-racial profiles at YDCs. Fifty-three percent of X-MEN respondents said they lived in precarious living conditions and severe material deprivation, which coincides with the identification of an urban profile of poverty and inequality in Portugal associated with high unemployment and extended households with children (Rodrigues, 2019). Similarly, the longitudinal study by Lansford et al. (2007) of 574 children followed from age 5 to age 21 to examine the potential links between early physical maltreatment and violent delinquency in late adolescence or early adulthood, showed that individuals who had been victims of physical maltreatment in the first 5 years of life were less likely to perform well academically and were at greater risk of being arrested as juveniles for delinquency and of becoming teenage parents, on the one hand; on the other hand, the child's race and gender moderated these links, since they were more prevalent in African-American youngsters and slightly more pronounced in female than in male.

While the differentiation of systems and spaces dedicated to Child Protection and Juvenile Justice in Portugal, legally determined in 1999, is often identified as an achievement of Portuguese public policies in guaranteeing the human rights of children and youngsters, the reality of their (lack of) articulation currently tells a very different story: according to X-MEN, the dominant perception of the YDC professionals, reinforced by the young people's accounts of their experiences in the foster care system, is that of a logic of facilitation and lack of supervision in the foster homes where the majority of these youngsters lived prior to the YDC.

At the time of the study, more than 90 percent of the young people surveyed were young people whom the State had failed to protect, and many said that they started offending precisely when they entered foster homes, where they learnt these practices. If there are families who wish their children well, they often do not know that their relationship with them is distorted, which leads to the child being removed and institutionalized under the protection system. However, placing a child or young person in foster care tends to aggravate their emotional ruptures and is characterized by a lack of supervision, relational distortions and a feeling of abandonment (Moura et al., 2024a).

The traumatic potential of foster care is further exacerbated when the child or young person enters the system more than once. According to public data (Instituto da Segurança Social (ISS), 2021),^5^ re-victimization resulting from re-entry into the foster care system has a higher incidence in males (55% in 2020). On the other hand, while the duration of foster care for younger age groups tends to be only for the time necessary to ensure their safe return to the family environment (on average, 1 year), for adolescents, the duration of this measure increases (on average, a minimum of 3 years since 2019) (Instituto da Segurança Social (ISS), 2023, 2022, 2021).

Given that pre-adolescence is marked by identification with their peers, control over young people's behavior by adults becomes challenging, which is even more difficult to exercise in an institutional setting, since there are mechanisms that Child Protection professionals cannot use (that are authorized under the TEA), such as the possibility of physical restraint.

## Gender as the basis for differentiated treatment of juvenile delinquency

When portraying shared individual factors for violence among at-risk youth in Portugal, Spain and Croatia, the X-MEN team identified, in line with several other authors (Wong et al., 2017; Levant and Richmond, 2007; Pleck et al., 1993), the impact of internalized gender norms and notions of hegemonic or dominant masculinity that portrayed “real men” as those who match social expectations of strength, honor, aggression, dominance and emotional stoicism (Slegh et al., 2021; Connell and Messerschmidt, 2005),

while vulnerability, sensitivity, and other traditionally “feminine traits may be stigmatized or suppressed” which can result in limited emotional expression or communication, pressure to engage in risk-taking or violent behaviors, and negative health outcomes such as substance abuse or mental health problems (Moura et al., 2024b, p. 10).

Research also shows that during childhood, boys tend to suffer more physical punishment than girls (Heilmann et al., 2021; Mahoney et al., 2000) and to be punished for behavior such as crying or whining, which is considered not to correspond to social expectations of masculinity (Sorbring et al., 2003). A study dveloped by the Johns Hopkins Bloomberg School of Public Health (Blum et al., 2019), for example, analyzing the potential link between adverse childhood experiences and violence perpetration or depressive sypmtoms, showed that boys are more likely to become violent in adulthood due to rigid gender norms, while girls tend to have higher levels of depression. The normalization of violent behavior and the exposure of children and young people to violence is also reflected in the predominance of male perpetrators and female victims of violent crime (Wolff et al., 2017). This phenomenon translates into the Portuguese YDCs, a highly structured and mostly male environment and where, as such, the models and practices of young boys often reflect rigid conceptions of masculinity and, more specifically, aggressive masculinity. In its population, there is an approximate ratio of 1 girl to 9 boys being held for committing offenses, with studies on female criminality with life trajectories showing not only that there tend always to be male figures behind female criminality, but also that judges do not criminalize women as much (Steffensmeier and Schwartz, 2004; Steffensmeier and Allan, 1996; Steffensmeier et al., 2023).

According to the combined analysis of the statistical data from the CASA^6^ (2023, 2022, 2021) and the CAFCE Reports (2023, 2022, 2021), reinforced by the X-MEN qualitative data, it can be concluded that both the Child Protection System and the Juvenile Justice System seem to be more lenient with girls, who they see as people, by definition, in need of protection and who, as such, are more often referred to the sphere of the Child Protection System; boys, on the other hand, are more easily seen as intentional perpetrators of these behaviors, without the same compassionate and protective gaze, so they tend to fall under the remit of the Juvenile Justice System and are more likely to be subjected to internment measures (the most restrictive): Data from the Portuguese Ministry of Justice describes boys as representing 83.8 percent (125 boys) of the total number of young people in detention, and the age of the majority is concentrated in the 16–17 age group. Looking at the statistical data on the capacity of YDCs according to the respective regime of execution of the measures, taking into account the youngsters' gender, it is possible to see how rare it is (13.5%) for the most restrictive regime of execution of such detention orders, i.e., the closed regime, to be applied to girls [Directorate-General for Reintegration Prison Services (DGRSP), 2024].

However, the lack of data that allows for a comparison between gender, previous interaction (or not) with the Child Protection System and the detention order applied does not allow for more in-depth conclusions to be drawn regarding the possible influence of this variable on the delinquent pathway, namely regarding the possible different treatment of young men and young women by both the Child Protection and the Juvenile Justice Systems.

## Challenges of intersectionality and trauma

In this respect, it is important to understand the intersectionality between dimensions such as gender, race, ethnicity, disability or behavioral problems and how subjects are socially controlled in different ways and establish different patterns of relationship with public authorities: with regard to young males in particular, behavioral issues or even those arising from socio-cognitive and emotional difficulties tend to be interpreted as deviant behavior (Moura et al., 2024a; Caruso and Paz, 2022).

In addition, boys and men of color are often viewed with fear, and are regularly not given the benefit of the doubt in the event of alleged bad behavior, which tends to be immediately associated with criminality (Wilson et al., 2017). This has significant consequences for the way these boys are treated by public authorities, both at school and in the justice system, and can reinforce an internalization of violence as a legitimate or even necessary way of creating some security in a hostile environment (Slegh et al., 2021).

This means that many of the male behavior that result from trauma derived of their adverse life trajectory is interpreted as delinquent, particularly in boys:

Professionals need to be aware of this racial trauma in boys and men, which often presents as deviant behavior, increased vulnerability to violence and abuse, vulnerability to rejection and accusation, increased self-awareness, feelings of being watched, judged, criticized and excluded (Michiels, 2024, p. 54).

At the extreme, the police are reconfigured in some national systems as instrument of discipline in the face of student behavior that does not require this extreme level of public authority (Swartz et al., 2016). This emphasis on permanent control and policing necessarily results in a disproportionate response to the behavior reported, leading to a significant increase in juvenile arrest rates. Richards and Cohen (2022), in a joint investigation between the Chicago Tribune and ProPublica, describe the case of a public school in Illinois, specializing in children and adolescents with disabilities, which frequently uses the local police to deal with situations that the education professionals there perceive as “indiscipline,” with half of the school's students being arrested in a single school year (2017-208) and officers arresting students (some as young as 9 years old) more than 100 times in the last five school years. The qualitative data from the X-MEN Project highlights this phenomenon, particularly in the context of the COVID-19 lockdown period between March 2020 and June 2021. During this time, young people increasingly perceived the police as agents of repression and violence rather than protection. This perception is exemplified by José (fictitious name), who, in an interview, acknowledged his involvement in gang fights but distinguished between fighting and aggression. In his view, aggression is perpetrated by the police, not by young people (José, X-MEN Participant, personal interview transcript, 2022).

Thus, if we look at the young people serving time in the 3 YDCs in Lisbon, we can identify the existence of a pattern, particularly in terms of geographical origin from a certain set of social neighborhoods configured as areas of greater social vulnerability. The fact that the young people remained on the streets, even during the compulsory lockdown decreed by Portuguese health authorities, exposed them even more to constant and surgical policing, which necessarily led to greater exposure of these youngsters to law enforcement agents who, due to compliance with the lockdown, were legitimized to search and approach them without any apparent reason for arrest. Thus, these young people, who were already at a disadvantage for living in poorer contexts and having worse health conditions, became doubly disadvantaged because they usually lived in less favorable housing that was too small for the size of their family, “forcing” them to spend as much time as possible on the streets (Caruso et al., 2023; Mascarenhas, 2024).

Several international monitoring organizations [European Committee for the Prevention of Torture Inhuman or Degrading Treatment or Punishment (CPT)., 2023; European Commission against Racism Intolerance., 2018], particularly within the Council of Europe, have consistently highlighted Portugal's ongoing challenges concerning police violence, disproportionately affecting Black and Roma populations. The ECRI, in its 2018 country report, documented numerous allegations of racially motivated violence perpetrated by law enforcement officials, accompanied by a systemic failure to conduct effective investigations. In a 2021 memorandum, the Council of Europe Commissioner for Human Rights, Mijatović (2021), urged Portuguese authorities to adopt a more robust and proactive approach to addressing the rise of racism, underlining the enduring nature of racial discrimination—especially against Roma communities, people of African descent, and individuals perceived as foreigners. The Commissioner also expressed concern regarding the prevalence of racist attitudes within police institutions, including reports of infiltration by far-right groups. More recently, in 2023, the European Committee for the Prevention of Torture reiterated its concerns over the persistent mistreatment of detainees by Portuguese police forces, documenting instances of physical abuse and the excessive use of restraints, and stressing the critical need for prompt and effective investigations as a deterrent mechanism.

From an intersectional perspective, it is essential to emphasize the increase in police hyper-surveillance and violence against youngsters based on their ethnicity. According to the field observation by researchers and focused interviews, it was possible to confirm that, in Lisbon's three youth detention centers, over 80% of young detainees are Black, and they frequently report experiencing police violence. It is not possible to obtain a reliable quantitative record of the ethnicity or race of the young people, as it is not legally permitted in Portugal to inquire about racial identity. However, some conclusions may be drawn from the fact that 20% of the participants who completed the questionnaire were born in Brazil, Cape Verde, Mozambique, Angola (X-MEN questionnaire data, personal communication, April 2022).

This trend reflects the concept of the adultification of Black children, as outlined in the State of UK Boys report (Equimundo, 2022). The report highlights that Black children are frequently perceived as more “adult-like”, less innocent, and less vulnerable compared to their white peers—perceptions rooted in the enduring legacies of slavery and colonialism (Bernstein, 2011; Walton, 2021). As a result, institutions may overlook their needs and fail to uphold their legal rights to protection and support. Consequently, Black children are disproportionately subjected to punitive measures, including strip searches in schools and police stop-and-search practices in their communities (Davis and Marsh, 2020; Equimundo, 2022, p. 8). The possible confirmation of such gender bias affirms the need to deconstruct gender prejudices and stereotypes concerning the commission of criminal offenses, given the way they are reproduced in the juvenile justice and protection systems, to minimize the harsher and more stigmatizing nature of the State's reaction to the delinquent behavior of young men, which makes it difficult to achieve the educational and rights-promoting purposes in equitable terms that this intervention should have.

## Final considerations

If adolescent *acting out* (acts of affirmation) explains a large part of episodic deviant behavior, the fact is that, to analyse these phenomena, additional research is needed, of an intersectional nature, which combines the analysis of the contributions of multiple factors that may influence the practice of these behaviors, such as ethnic-racial profiles, constructions of masculinities and femininities, power relations, the influence of territories (e.g., the impact of excessive policing), among others.

It is, therefore, essential to underscore the critical role of prevention in mitigating juvenile delinquency and crime (Tillyer et al., 2011), particularly given the absence of a comprehensive public system or policy designed to address paradelinquency—a category of behaviors that fall outside the remit of the child protection system, the educational guardianship system, let alone the criminal justice system. Furthermore, it is imperative to invest in intermediate structures that facilitate the (re)integration of young boys and girls into society following the completion of their protective or correctional measures. Equally important is the implementation of consistent and sustained interventions involving both the families and social environments of these youths, aimed at creating conditions conducive to the development of alternative life trajectories. Such interventions are pivotal in preventing recidivism, as reintegration into unchanged familial and social contexts may undermine the rehabilitative efforts accomplished within institutional settings, as reflected in the interviews with professionals in all the 6 Portuguese youth detention centers. The X-MEN data, although in the context of a small study (Mears et al., 2015), seems to indicate the possibility of a gender bias in the application of Juvenile Justice in Portugal, based on a more repressive view of boys and a perception of greater victimization of girls.

In an era marked by evolving notions of masculinity and a greater awareness of social justice, the exploration of gender identities to assert their implications for peaceful coexistence has become imperative. Traditional notions of masculinity often perpetuate harmful stereotypes and contribute to widespread gender-based violence and discrimination, and children, especially boys, are socialized to adopt risky and domineering behaviors. This reality is all too evident in adolescence and often reveals itself in delinquent acts that lead to young people being sent to detention centers. In this sense, there is an urgent need to promote healthy alternatives to these models of masculinity in hierarchical contexts where the aim is to re-socialize young people, as is the case with YDCs.

These detention facilities have their own organizational culture; however, efforts must continue to ensure that their legal and intervention framework is geared toward the best interests of young vulnerable boys and girls. This means adopting an intervention philosophy in which restorative justice practices replace punitive ones (Moura et al., 2024b; Terra, 2009). This is all the more important given that the juvenile justice system's efforts to stimulate young people's agency are based on seemingly artificial changes in behavior that conversely appear to continue to perpetuate their vulnerability and alienation:

(…) the “wholly individualized fully competent subject” (Ruddick, 2007: 638; see also Viego, 2007) envisaged by juvenile justice systems is a fiction, and that young people's aspirations for wholeness may result in their domination by the behavioural change practices which are said to liberate them (Cox, 2011, p. 604).

In order to counteract the effects of a socialization based on models of dominant and aggressive masculinity, and to provide alternative models of more equitable, caring and healthy masculinities, “reworking masculinities into identities of care rather than domination” (Elliot, 2016, p. 240), YDC professionals should be prepared to detect and overcome their own prejudices and biases when working with vulnerable boys and girls (Lubben, 2019). Their initial and ongoing training should be strengthened, covering themes such as gender and intersectional perspectives, notions of developmental psychology, trauma, conflict management and mediation to enable such professionals to work with young people based on their specific needs (Longo et al., 2024; Moura et al., 2024c).

A comprehensive analysis of how multiple social categories—such as race, gender, class, and migration status—intersect in shaping young people's experiences of violence is fundamental to a deeper understanding of the structural inequalities that underpin these dynamics. This intersectional approach is essential for the development and implementation of interventions that are not only attuned to individual circumstances but also capable of fostering structural change by challenging the broader systems of oppression that sustain social disparities. Acknowledging that we operate within a structurally inequitable society, our efforts are directed toward deconstructing these interwoven forms of inequality as they materialize in the lived experiences of each young person.

The potential of comparative institutional studies in understanding diverse approaches to youth justice systems is both compelling and necessary. Grossi's (2021) critical analysis of Brazil's educational model of social reintegration, for example, reveals both the promises and the structural constraints inherent in models intended as alternatives to incarceration. His ethnographic and documentary research highlights the potential for reintegration frameworks that move beyond bureaucratic rhetoric, even in contexts marked by profound social and economic challenges. Yet, Grossi also underscores the persistent challenges of inclusivity and reintegration in societies marked by structural inequalities and economic instability. While the X-MEN project did not encompass comparative analysis, it is clear that future research should extend this inquiry to include alternative models, such as restorative justice frameworks, which have demonstrated promising practices in other international youth justice systems. Such comparative studies could offer valuable counterpoints to the Portuguese YDC model, fostering critical reflection on how educational and reintegrative principles might be effectively reimagined in different contexts. One such model, the APAC (Association for the Protection and Assistance of Convicted People), represents an innovative approach grounded in community involvement, education beyond traditional schooling, and active participation by prisoners, volunteers, and external institutions [Fraternidade Brasileira de Assistência aos Condenados (FBAC)., 2019]. The global expansion of APAC underscores its potential to inform public policy and transformative practice.

While our project did not encompass comparative analysis, we acknowledge its crucial importance and, in our conclusion, highlight the need for future research to examine alternative frameworks, including restorative justice approaches, as counterpoints to the Portuguese YDC model.

By challenging conventional norms and encouraging a more inclusive and understanding masculinity, based on empathy as a fundamental value, it becomes easier to facilitate transformative changes in interpersonal relationships, community dynamics and wider social structures and contribute to the secondary prevention of violence. We argue, therefore, for professional training informed by trauma sensitivity and gender considerations, the creation of intermediate reintegration structures, and the development of care systems that prioritize individual dignity over control and fear. Crucially, future research should explore whether these proposals can be operationalised without perpetuating new forms of institutional paternalism.

To this end, further research is needed, of an intersectional nature, combining the analysis of the contributions of multiple factors that may influence the practice of delinquent behaviors, such as ethnic-racial profiles, constructions of masculinities and femininities, power relations, the influence of territories (e.g., the impact of excessive policing), among others.

## Data Availability

The datasets presented in this study can be found in online repositories. The names of the repository/repositories and accession number(s) can be found below: https://xmen.ces.uc.pt/.

## Footnotes

1 Annual Reports from the Youth Detention Centres Monitoring and Inspection Commission.

2 Commission for the Integrated Analysis of Juvenile Delinquency and Violent Crime.

3 Law No. 166/1999, of 14 September – Tutelary Educational Act. Available at Lei Tutelar Educativa | DR (diariodarepublica.pt).

4 Law No. 147/1999, of 1 September – Law for the Protection of Endangered Children and Young People. Available at Lei n.° 147/99 | DR (diariodarepublica.pt).

5 Social Security Institute.

6 Report on the Annual Characterisation of the Fostering Situation of Children and Youngsters in Portugal.
